# Effect of Strain and Surface Proximity on the Acceptor Grouping in ZnO

**DOI:** 10.1021/acsomega.3c06556

**Published:** 2023-11-01

**Authors:** Oksana Volnianska, Vitalii Ivanov, Lukasz Wachnicki, Elzbieta Guziewicz

**Affiliations:** Institute of Physics, Polish Academy of Sciences, 02-668 Warsaw, Poland

## Abstract

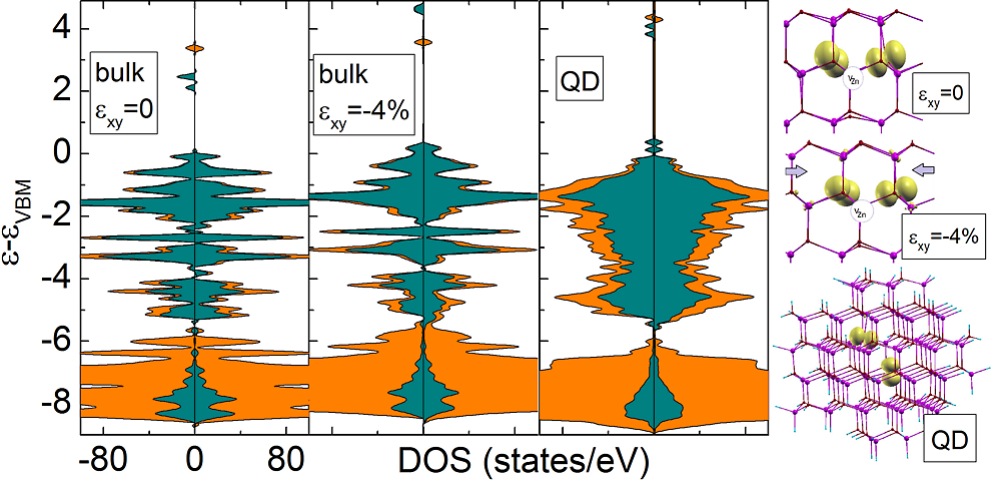

According to the present knowledge, the level of zinc oxide conductivity is determined by donor and acceptor complexes involving native defects and hydrogen. In turn, recently published low-temperature cathodoluminescence images and scanning photoelectron microscopy results on ZnO and ZnO/N films indicate grouping of acceptor and donor complexes in different crystallites, but the origin of this phenomenon remains unclear. The density functional theory calculations on undoped ZnO presented here show that strain and surface proximity noticeably influence the formation energy of acceptor complexes, and therefore, these complexes can be more easily formed in crystallites providing appropriate strain. This effect may be responsible for the clustering of acceptor centers only in certain crystallites or near the surface. Low-temperature photoluminescence spectra confirm the strong dependence of acceptor luminescence on the structure of the ZnO film.

## 1 Introduction

The experimentally observed two types of photoelectron spectra (PES) coming from different crystallites in ZnO and ZnO/N suggest that the grouping of acceptor and donor complexes occurs in separate domains.^1^ Density functional theory (DFT) calculations reveal that the complexes involving zinc vacancy (*V*_Zn_), hydrogen (H_*x*_), and, in the case of ZnO/N, also nitrogen modify the density of states (DOSs) of the valence band maximum (VBM). This phenomenon translates into the experimentally observed differences in PES between crystallites containing different acceptor-related complexes.^1^ According to a number of experimental and theoretical works, native defects and their complexes, often containing hydrogen, such as *V*_Zn_, *V*_O_, *V*_Zn_–*n*H (*n* = 2, 3), Zn_i_*V*_O_H, and others, determine ZnO conductivity as they introduce shallow and deep, donor and acceptor levels,^2−11^ and the acceptor-related sample properties depend on a number of factors introduced by growth and/or annealing conditions.^12−15^ For example, for ZnO/N it has been shown that both annealing medium and temperature influence acceptor-related cathodoluminescence intensity, which is higher under oxygen annealing.^15^ Generally, the reasons for donor and acceptor domains are not clear, but it might be due to the formation of defect clusters that require distortion of the crystal lattice. Hence, it can be assumed that microstrain plays a role here.

In fact, strain can significantly modify the electronic structure of a semiconductor, thus it can have a strong effect on its structural, electrical, and optical properties.^16−20^ For example, hydrostatic pressure-induced redistribution of native defects and the optoelectronic response have been indicated in ZnO rode-like nanocrystals.^21^ Although for ZnO films and single crystals the strain effect has been less studied, for strongly correlated oxides it has been demonstrated that a wide spectrum of technologically relevant functional properties can be tuned via strain.^22−25^ In particular, it was indicated that tensile strain generally favors the formation of anion vacancies in such materials, and compressive strain promotes cation vacancies.^22−24^ In most experimental studies, the physical properties of ZnO under uniaxial or biaxial stress/strain have been investigated from the point of view of epitaxial thin films or nanocrystals.^16−21,26,27^ In particular, the effect of substrate-induced strain in ZnO thin films on different substrates has been investigated by X-ray diffraction and photoluminescence measurements, and the excitonic peak positions are found to shift slightly toward the lower energy side with increasing uniaxial strain.^28^ Also, despite extensive theoretical studies of ZnO, research interest has mostly focused on the strain effects affecting the band gap and the electronic structure of pure ZnO,^18,29−33^ the magnetic and electronic properties of the doped system,^30,34−36^ or native defects under extremely high pressure.^37^

In refs (1) and (38), we have carried out some study of the electronic structure of Zn vacancy in ZnO. Here, using first-principles band structure calculations, we investigate the electronic band structure of ZnO/H, especially the electronic structure and formation energy of H interstitial, Zn vacancy, and *V*_Zn_H complex in ZnO as a function of different strain conditions. We model the observed acceptor domains as simple 0D nanocrystals with a passivated surface, i.e., quantum dots (QDs). Thus, we study here the surface proximity of these defects in QDs. Our results elucidate the effects of strain and surface proximity on the electronic structure and grouping of vacancy–hydrogen complexes. In particular, we demonstrate that strain leads to a decrease of the acceptor complex formation energy, so it might be responsible for the grouping of acceptors, which can only form in crystallites showing compressive strain or if they are close to the surface. In support of the DFT calculations, we present the results of photoluminescence measurements, which reveal considerably different acceptor luminescence for ZnO layers deposited on *c*– and *a*– oriented sapphires, i.e., showing a different crystallographic structure.

## 2 Methods

### 2.1 Computational Methods

Calculations based on the density-functional theory (DFT) within the generalized gradient approximation (GGA)^39,40^ were performed using the QUANTUM-ESPRESSO package.^41^ On-site, Hubbard-like + *U* terms^41,42^ were applied to the d(Zn) and p(O) orbitals as *U*_Zn_ = 10 eV and *U*_O_ = 7 eV,^1,43,44^ reproducing to within 1% the experimental lattice parameters of wurtzite (*w*) ZnO (*a*_0_ = 3.223 Å and *c*_0_ = 5.24 Å), the experimentally established band gap, *E*_g_, at about 3.38 eV and the energy of the core *d*(Zn) level, centered about 7.5–8.4 eV below the VBM.^45,46^ Figure S1 in Supporting Information shows the DOS of a perfect ZnO crystal calculated for the above + *Us* parameters. Ultrasoft atomic pseudopotentials^39^ were employed, and the following valence orbitals were chosen: 3d^10^ and 4s^2^ for Zn, 2s^2^ and 2p^4^ for O, 2s^2^ and 1s^1^ for H. The plane wave basis with the kinetic energy cutoff of 40 Ry provided a good description of II–VI oxides. The Brillouin zone summations were performed using the Monkhorst–Pack scheme with a Γ point and 2 × 2 × 2 *k*-points mesh.^47^ Ionic positions were optimized until the forces acting on ions were smaller than 0.02 eV/Å both in the bulk for the 128-atom supercell and QDs structures. The smearing width of 0.136 eV in the Methfessel–Paxton method was chosen to account for partial occupancies and to guarantee convergence and the lowest supercell total energy.^48^

We focused on two types of strain conditions: (1) the biaxial strain, i.e., in the *xy*-plan; and (2) the uniaxial strain applied along the *c*-axis direction. We defined the biaxial and uniaxial strains as ε_*xy*_ = (*a* −*a*_0_)/*a*_0_ × 100% and ε_*zz*_ = (*c* – *c*_0_)/*c*_0_ × 100%, respectively, where *a*_0_, *c*_0_ and *a*, *c* are the lattice constants of the perfect single crystalline ZnO in its equilibrium and strained states, respectively. Thus, in the tensile and compressive cases, ε_*xy*_ and ε_*zz*_ are positive and negative values, respectively. For example, biaxial strain ranging from −4 to 4% was realized by compressing or stretching the material in the *xy*-plane, i.e., during the calculations, the lattice parameters were kept fixed at the specified strained *a* = *b* and *c* = *c*_0_ and the internal coordinates of atoms were relaxed. Additionally, considering the substrate clamping effect, we performed some calculations with fixed *a* = *b* for each strain, while relaxing the *c*-axis length; however, in both cases the results were qualitatively similar (see Supporting Information, Section S1, Figures S2–S8).

Approximately spherical Zn_84_H*_57_H**_57_O_84_ QDs (a diameter of about 15 Å) containing 168 host atoms and 114 pseudo hydrogens were constructed using the bulk wurtzite crystal structure provided that no more than two dangling bonds were left on the surface, in agreement with our previous study^44^ and other works.^49^ To isolate the QDs and avoid inter-QD interactions, a vacuum spacing of ∼15 Å was taken. Dangling bonds of Zn^2+^ and O^2–^ ions on the uncompensated surface were passivated by pseudohydrogen atoms H* and H** with fractional charges, +1.5 and +0.5, respectively.^44,49,50^ The Zn–H* and O–H** bond distances were taken from optimized ZnH_4_ and OH_4_ tetrahedra. After relaxation, the length of Zn–H* is 1.6732 Å and that of O–H** is 0.98 Å. The finally optimized geometry of the undoped QD deviates from the initial bulk wurtzite structure. In the vicinity of Zn atoms in the QD center, the tetrahedral wurtzite *C*_3*v*_ symmetry is maintained. However, the optimized Zn–O bond lengths near the surface are about 1.5% shorter than those inside the QD (relaxed structure of the QD is shown in Figure S9, Supporting Information). We apply the *U*_Zn_ = 10 eV and *U*_O_ = 7 eV values to the QD calculations,^44^ and we obtained *E*_g_ as 4.4 eV that is in full agreement with the ZnO QDs experimental results.^51^ Figure S10 in Supporting Information shows the calculated DOS of QDs for above + *Us* parameters.

### 2.2 Synthesis of ZnO Thin Films

ZnO films investigated here were grown by atomic layer deposition (ALD) using diethylzinc and water precursors. The details of the growth process can be found elsewhere.^13^ About 120 nm thick films were deposited simultaneously on *c*-Al_2_O_3_ and *a*-Al_2_O_3_ substrates in the same ALD process. As previously shown, deposition on differently oriented sapphire substrates strongly influences orientation of ZnO films over a wide growth temperature range, especially for limited layer thicknesses.^15^ The studied layers were deposited at a temperature of 300 °C, which is high for the ZnO–ALD process, in order to get a high-intensity photoluminescence signal despite the limited layer thickness. Rapid thermal annealing (RTA, 800 °C, 3 min) in oxygen atmosphere was applied in order to further enhance layer quality and to facilitate the formation of zinc vacancies. As previously shown, ZnO samples grown by ALD have a high hydrogen content that after RTA was reported at the level of 10^19^/cm^3^.^12,15^ For this reason, hydrogen atoms are included in the theoretical calculations.

### 2.3 Photoluminescence Measurements

Photoluminescence (PL) spectra of ZnO samples were studied in the range of temperatures 5–100 K. Samples were mounted in an optical cryostat with a variable temperature exchange gas flow. Temperature stability during the experiment was kept at the level 0.05 K. Samples were excited by the third harmonic of a Nd^3+^ YAG laser with a photon energy of 3.493 eV and a power density of 5 W/cm^2^. For detection of PL spectra, a spectrograph/monochromator with a 0.28 m grating equipped with a Hamamatsu C7042 thermoelectric charge-coupled device camera was used.

## 3 Results and Discussion

**Figure 1:**
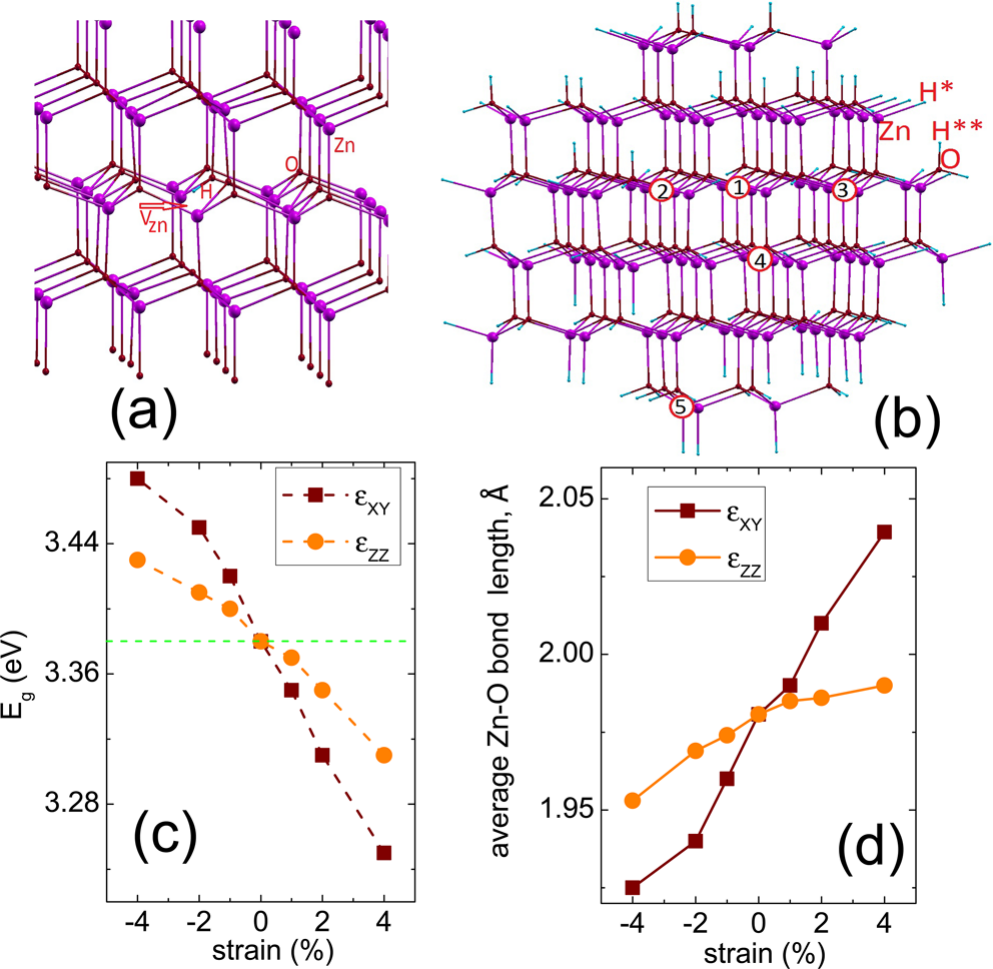
(a) Relaxed structure of V_Zn_H in the unstrained ZnO. (b) Relaxed structure of the QD. Purple and red balls represent Zn^2+^ and O^2–^ ions, respectively. Smaller cyan balls denote pseudohydrogen atoms (H* and H**) employed for surface passivation. The numbers 1–5 denote, respectively, the Zn (or vacancy) sites: 1: *c*, 2: *m*_1_, 3: *m*_2_, 4: *m*_3_, and 5: *m*_4_. (c,d) Variations of the energy of the band gap (c) and average Zn–O bond length (d) under different strains for ZnO bulk.

We consider a bulk supercell and a QDs crystal containing various point defects, including interstitial hydrogen (H_*i*_), Zn vacancy (*V*_Zn_), and (*V*_Zn_–H) and (*V*_Zn_–H_2_) complexes. Hydrogen interstitial is suggested to have a donor-like ground state in ZnO at a bond-center site,^1,52,53^ and in this work, we compute H_*i*_ in this configuration. Figure 1a shows optimized atomic structure for *V*_Zn_–H in the bulk ZnO. We also calculate the electronic structure of the *V*_Zn_ and *V*_Zn_–H complexes at four different positions in the QD: near the QD center and at three midway sites. Below, we refer to these sites as *c*, *m*_1_, *m*_2_, and *m*_3_, respectively. The QD relaxed geometry and the precise placement of the vacancy sites are presented in Figure 1b. We do not consider the vacancy at *m*_4_ sites because it is a surface site, and in this case the surface properties are strongly dependent on the passivation species. Thus, in all midway positions, likewise for the center site, the vacancy is surrounded by four O atom neighbors. The total energy of the H_*i*_ interstitial was calculated for nine different positions in the QD (see Figure S11, Supporting Information). For each Zn at *c–m*_4_ sites, hydrogen was introduced at the center of Zn–O bond in the *xy*-plane and at the bond along the *c*-axis.

The effect of strain conditions on the ZnO band gap and defect formation energy was investigated by calculating the total energies for the defective system and for pure ZnO under different ε_*xy*_ and ε_*zz*_ in the range between −4% compressive strain and 4% tensile strain, respectively. Figure 1c shows the effect of strain on the band gap value of pure ZnO at the Γ point. The *E*_g_ curve versus strain follows a sublinear relationship regardless of the strain direction. With the increasing compressive (tensile) strain, the *E*_g_ value increases (decreases) about 0.11 (0.13) and 0.06 (0.07) eV for ε_*xy*_ and ε_*zz*_, respectively. The trend of the *E*_g_ curve in our calculations is consistent with that in other reports.^29,31,35,36,54^ Interestingly, experiments have shown that the values of the ZnO band gap ought to be corrected according to the Burstein–Moss effect also based on the sublinear relationship.^33^ Under compressive (tensile) strain, the length of the Zn–O bond is shortened (elongated) (see Figure 1d), which leads to enhanced (diminished) overlapping of electron orbitals. Because both the VBM and the conduction band minimum (CBM) are antibonding states related to the d(Zn)–p(O) and s(Zn)–s(O) hybridization, respectively; thus, under compressive (tensile) strain, the VBM and the CBM are shifted down (up) with respect to the unstrained case. However, the shift of the VBM is higher, so we observe that the band gap widens (narrows).^18,55^

### 3.1 Formation Energy under Strain

For all configurations, we calculate the defect formation energy *E*_form_ as^56,57^
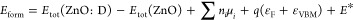
1where *E*_tot_(ZnO/D) and *E*_tot_(ZnO) are the total energy of the supercell with and without the defect (or complex), respectively; n_*i*_ is a number with the + (−) sign corresponding to the removal (addition) of atoms, μ_*i*_ is the variable chemical potential of atoms in the solid, *q* is the charge state of defect (or complex), ε_F_ is the Fermi energy referenced to the VBM, ε_VBM_ is the energy of the VBM of ZnO, determined in line with the algorithm in ref (57), and E* is the finite size supercell correction, including the potential alignment correction of the VBM and the image charge correction.^57^ We did not include E* in the QDs calculations. In line with our experiments,^1,15^ the O-rich experimental conditions are considered. Thus, we calculate μ_*i*_ as μ_Zn_ = *E*_tot_(Zn bulk) + Δ*H*_f_(ZnO), μ_O_ = *E*_tot_(O_2_)/2, and μ_H_ = *E*_tot_(H_2_)/2 + 1/2 Δ*H*_f_(H_2_O). Here, *E*_tot_(Zn bulk), *E*_tot_(O_2_)/2, and *E*_tot_(H_2_)/2 are total energies per atom of the elemental solids. Δ*H*_f_ is the enthalpy of formation per formula unit, which is negative for stable compounds. Δ*H*_f_ at *T* = 0 K is obtained by considering the reaction of formation or decomposition of a crystalline from its components.

**Figure 2:**
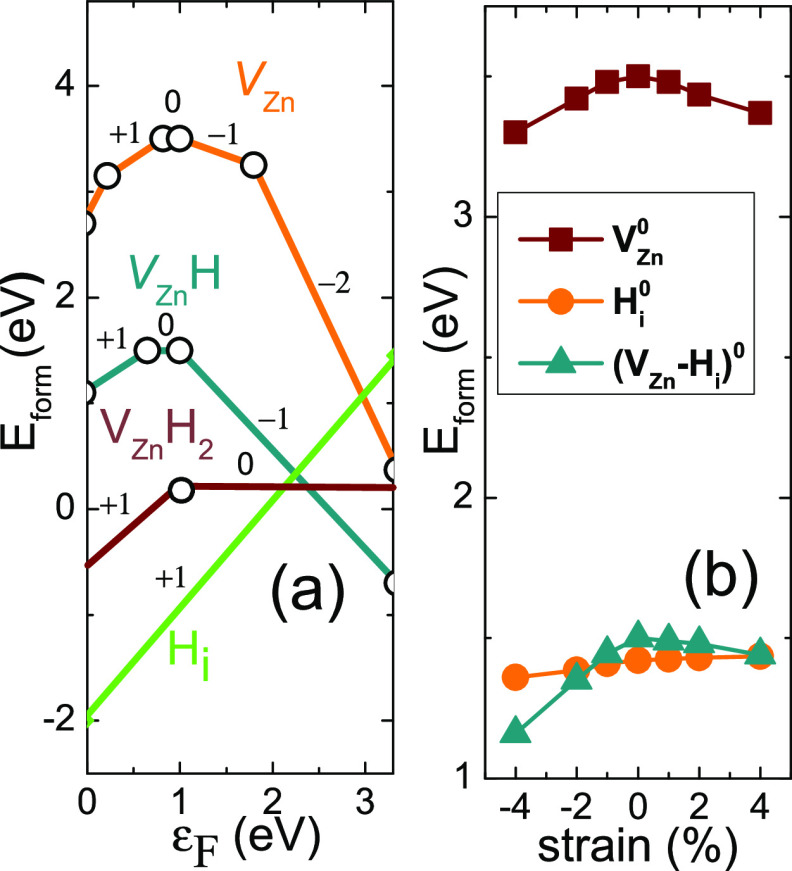
(a) Defect formation energies of unstrained ZnO as functions of ε_F_. (b) Variation of the *E*_form_ of *V*_Zn_, H_*i*_^0^ and  on the biaxial strain.

The results of the calculations are shown in Figure 2 and Table 1. In particular, Figure 2a shows the defect formation energies obtained using eq 1 for conditions without strain. The numbers near the lines correspond to the defect charge states (*q*), while the circle points represent the thermodynamic transition levels ε(*q*_1_/*q*_2_) defined as the ε_F_ at which the defect *E*_form_ in the *q*_1_ and *q*_2_ states are equal.^57^ If the calculated ε(*q*_1_/*q*_2_) lies in the band gap, we perceive it as a stable state, in contrast to the defect levels that are resonant and located below the VBM or above the CBM.

**Table 1:** Defect Formation and Complex Binding Energies, in eV, in the Bulk under Biaxial Strain and at the Different Sites in the QD

	bulk							QD			
defect	–4%	–2%	–1%	0	1%	2%	4%	*c*	*m*_1_	*m*_2_	*m*_3_, *m*_4_
H^0^	1.36	1.385	1.41	1.42	1.425	1.43	1.435	–0.182	–0.19	–0.125	–0.05, −0.31
V_Zn_0	3.3	3.42	3.48	3.5	3.48	3.435	3.35	2.8	2.8	2.85	2.55
(*V*_Zn_H)^0^	1.16	1.35	1.445	1.5	1.49	1.485	1.46	0.54	0.56	0.59	0.49
*E*_b_(V_Zn_H)	3.5	3.455	3.445	3.42	3.415	3.38	3.325	2.08	2.05	2.135	2.01

From Figure 2b and Table 1, we observe that in the investigated range of strain values, the *E*_form_ curve versus strain for hydrogen interstitial follows a sublinear relationship, and as the compressive strain increases from 0 to 4%, the *E*_form_ decreases from 1.42 to 1.36 eV. In contrast, increasing tensile strain causes the *E*_form_ of H^0^ to increase from 1.42 to 1.45 eV. With the introduction of H into the host, the total number of electrons in the system increases, which induces compressive stress in the crystal lattice. This trend is similar to the results from ref (58), but it should be noted that in the latter case very small supercells were used, which translated into very high H concentrations.

The formation energy of both zinc vacancy and the (*V*_Zn_H) complex is not a monotonic function of the applied strain, and an increase of any strain leads to a decrease in *E*_form_ but for the compressive strain the effect is stronger. In particular, the changes in *E*_form_ are about 0.12 and 0.34 eV in the case of 4% compressive strain, for *V*_Zn_ and (*V*_Zn_H), respectively. Similar variations in the formation energy for *V*_Zn_ were observed in refs (35) and (59). In particular, ref (35) demonstrated for the first time that *E*_form_ of zinc vacancy does not depend linearly on the strain due to the large structural relaxation around the vacancy.

The binding energy of the complexes, *E*_b_(*V*_Zn_H), was evaluated as the difference in the formation energy between the isolated constituents of the complex and the complex itself for a given Fermi level position. The positive *E*_b_ values indicate the energetic preference for the defect complex to be formed. Results are shown in Table 1. In our simulations, the presence of H_*i*_ is responsible for increased stability, i.e., a reduction of the formation energy of the *V*_Zn_ point defect. *E*_b_ = 3.42 eV for unstrained ZnO, and this value is increased by about 0.08 eV when the compressive strain increases to 4%. In contrast, the stability of the (*V*_Zn_H) complex decreases with increasing tensile strain. Figure 2a shows that the *V*_Zn_H_2_ complex has the lowest *E*_form_ = 0.26 eV and at the same time the highest *E*_b_ = 2.66 eV with respect to *V*_Zn_H and H_*i*_.

From a very simple model, the *E*_form_ decreases as the crystal is strained along the direction of strain induced by the defect.^35^ Thus, from this point of view, if a defect gives rise to a reduction of the local crystal volume, the external compressive strain leads to a lower formation energy compared to that of the material without strain. The change in the total energy of point defects under strain is determined by the size of the defect-induced local volume change and can exhibit either parabolic or superlinear behaviors.^60^ However, it needs to be noted that both *E*_form_ and electronic properties are affected by a lot of other factors such as defect electronic environment,^35^ symmetry, spin properties, and surface proximity, just to name a few. In particular, for example, ref (38) indicated that interatomic distances (given by the lattice constant and atomic relaxations around the cation vacancy) determine both the defect electronic structure and the stability of spin-polarized states in a number of III–V and II–VI semiconductors. Introduction of hydrogen or *V*_Zn_ into the lattice leads to large atomic perturbations. The increase in the energetic stability of vacancy and complexes with increasing strain can be explained by the distortion effects in the crystal structure due to the large atomic displacement during the relaxation process. Moreover, the observed structural distortions are complex; although the displacements of the second and further neighbors of defects are an order of magnitude smaller, the effect of atomic relaxations around the vacancy, involving not only the nearest but also more distant neighbors, cannot be neglected. The calculated relaxation energy is more than 1.5 eV for *V*_Zn_ under 4% strain.

Finally, we calculated the formation energy of the H interstitial *V*_Zn_ and its complexes at different positions in the QDs. In contrast to the bulk crystal, in the case of QD, we obtained very close total energies for hydrogen geometries in the *xy*-plane and bond center along the *c*-axis. Moreover, in some configurations the energy in the latter case was lower than it was in the basal plane. We note that in all cases the position of hydrogen with respect to the QD center plays a dominant role. At the same time, the energy difference between the basal plane and the *c*-axis was less than 6 meV; hence in Table 1, we provide only values of the lowest energies of H located either in the center of the axial bond or along the *c*-bond. Interestingly, the calculated length of the relaxed H–O bond was about 0.96 Å in all configurations. We note that the same bond length, 0.96–0.98 Å, was obtained for all bulk systems, both passivated H**–O on the QD surface and the one optimized for OH_4_ tetrahedra.^44,49^ According to the results of Table 1, one can see that H-doping in the QD is energetically favorable compared to that in the bulk crystal. Moreover, the lowest *E*_form_ value was obtained for H at a location near the QD surface (*m*_4_). We calculated the average Zn–O bond lengths for Zn at the *c*–*m*_4_ positions in the relaxed QD without defects. They are 1.9831, 1.9829, 1.9837, 1.9883, and 1.9653 Å for *c*, *m*_1_, *m*_2_, *m*_3_, and *m*_4_, respectively. The obtained values combined with the results from Table 1 give rise to the conclusion that as the length of the Zn–O bond decreases, the formation energy of H, which passivates this bond, decreases as well. The lowest *E*_form_ value was obtained for H at the *m*_4_ site. This agrees well with the bulk results, which demonstrate a reduction in formation energy under low compressive strain. Moreover, the above observation may explain why, in the QD calculations, H favors the Zn–O center along the *c*-axis in contrast to the bulk crystal. We obtained that in the relaxed QD structure, the Zn–O bond lengths parallel to the *c*-axis are shorter than those vertical to the *c*-axis.

The formation energy of Zn vacancy at the *c*-site of the QD is approximately 0.7 eV lower than in bulk ZnO (see Table 1). Moreover, *V*_Zn_ at this site is more favorable than that at the *m*_1_ or *m*_2_ sites. However, the lowest *E*_form_ value was obtained for the *m*_3_ site. Similarly, the formation energy of the (*V*_Zn_H) complex at these points of the QD is approximately 1 eV lower than that of bulk ZnO. A similar decrease in the formation energy of zinc vacancy and the (*V*_Zn_H) complex compared to the bulk calculations was also observed for the two-dimensional surface in ZnO.^61^

### 3.2 Defect Electronic Structure under Strain

In bulk unstrained ZnO, the calculated ε(+1/ −1) level of H_*i*_ is 3.31 eV and is resonant with the conduction band, which assumes a shallow donor level. Such a shallow level inevitably introduces a local deformation of the CBM around the defect. Figures S1 and S3 (see Supporting Information) show the DOS results for pure bulk ZnO and ZnO/H_*i*_. The VBM of both crystals is formed mostly from *p*(O) orbitals, while the CBM is built from *s*(Zn) and *s*(O) states. However, in the case of ZnO/H_*i*_, a significant contribution of the *s*(H) states to the CBM is also witnessed.

**Figure 3:**
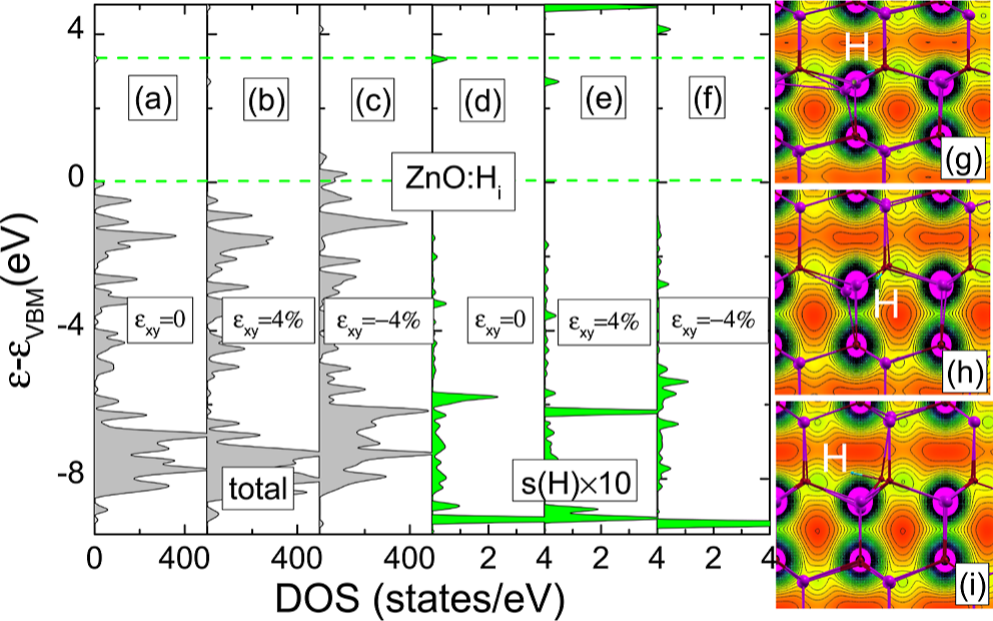
(a–c) Total DOSs of ZnO/H_*i*_ for unstrained and 4% tensile and compressive strained crystal, respectively. (d–f) Contribution (×10 times) of s(H) orbital for unstrained and 4% tensile and compressive strained crystal, respectively. Green dashed horizontal lines correspond to the VBM and CBM unstrained crystal. (g–i) Atomic configuration and the density of charge states of unstrained and 4% tensile and compressive strained crystal, respectively. Contours go from 0.0009 to 0.5 electron/Bohr^3^.

As in pure ZnO, the VBM and the CBM of ZnO/H_*i*_ shift down or up with respect to the unstrained system under tensile or compressive strain, respectively. Figure 3 shows the calculated total DOS and the contribution of s(H) orbitals of unstrained ZnO/H_*i*_ in comparison with the results under 4% tensile and −4% compressive strain conditions. We can see that under tensile strain, H states associated with the CBM move deeper into the gap with respect to the level of the unstrained system. The electronic structure is affected by atomic displacements around H (see Figure 3g–i). Interstitial hydrogen becomes the most stable when forming a bond with oxygen. The H–O bond length changes from 0.985 to 0.96 Å for different strain values ranging from 4% tensile to 4% compressive, respectively. In the latter case, we obtained the lowest *E*_form_.

**Figure 4:**
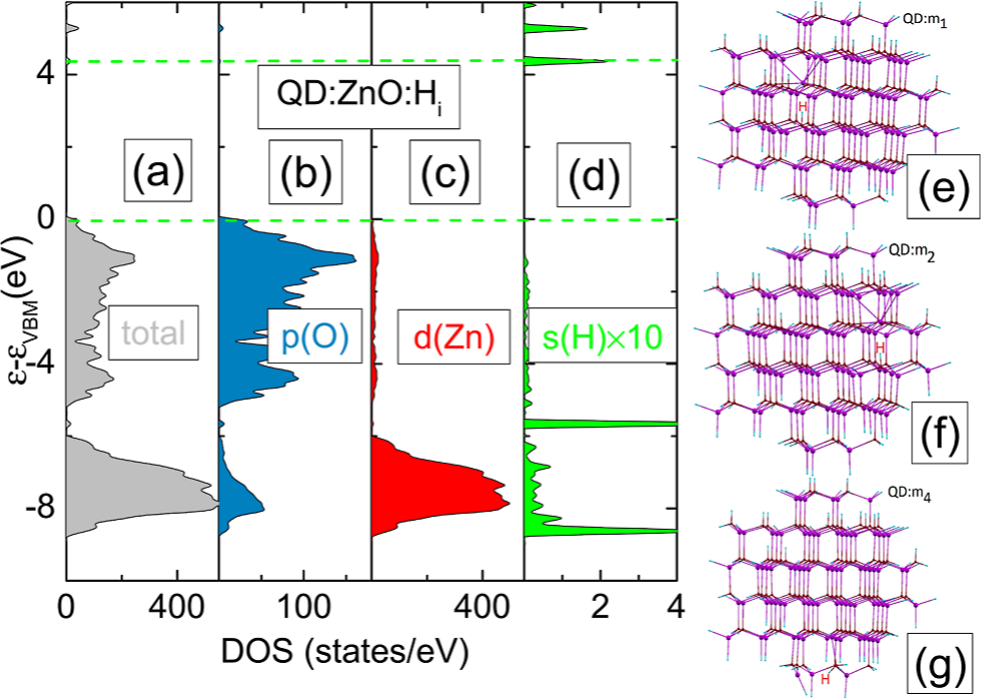
(a) Total DOS and (b–d) contributions of p(O), d(Zn), and s(H) orbitals of QD ZnO with H_*i*_ at *m*_1_ site. Horizontal dashed lines correspond to the VBM and CBM states. (e–g) Relaxed crystal structure of QD with H interstitial in the *m*_1_, *m*_2_, and *m*_4_ configurations, respectively.

Figure 4 shows the calculated DOSs for H_*i*_ in the QD crystal. As for bulk ZnO calculations, H_*i*_ states are associated with the conduction band states and are pinned to the CBM at all sites due to the shallow H_*i*_ shallow character. H interstitial in the QD also forms a strong bond with oxygen atoms, giving rise to large structure relaxations of the surrounding atoms (Figure 4e–g). The average O–H bond length (0.96 Å) is slightly shorter than in the case of bulk ZnO (about 0.98 Å). The strength of the O–H bond is a key factor in stabilizing the defect configuration; therefore, for higher compressive strain, a further decrease in the formation energy can be expected.

**Figure 5:**
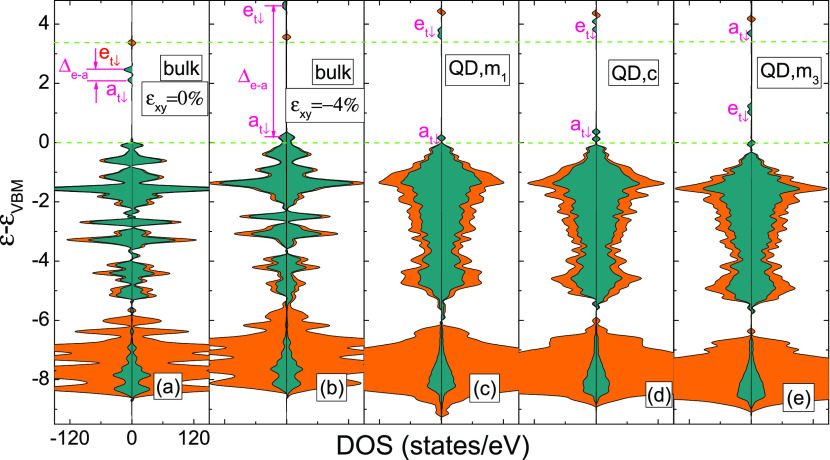
(a–e) Total (orange color) and the contribution of p(O) orbital (dark cyan color) spin-resolved DOS of ZnO containing a *V*_Zn_: (a,b) bulk under 0 and 4% compressive strain, respectively. (c–e) QD with vacancy at the *m*_1_, *c*, and *m*_3_ sites, respectively. Left and right panels denote the spin-down and -up channels, respectively.

In wurtzite ZnO, the *V*_Zn_—induced states can be considered as a combination of four dangling bonds of four O atoms surrounding the vacancy. H_*i*_ is located at one of the four dangling sp^4^(O) bonds when *V*_Zn_ is passivated by hydrogen. Four sp^4^(O) orbitals of oxygen neighbors combine into a singlet and a quasi triplet (a pair of doublet *e*_*t*_ and singlet *a*_*t*_) that are higher in energy. The quasi triplet is occupied with 4 or 5 electrons in the case of neutral *V*_Zn_ or the complex, respectively. The electron–electron exchange interaction splits the *e*_*t*_ and *a*_*t*_ levels, setting them into spin-up (*e*_*t*↑_, *a*_*t*↑_) and spin-down (*e*_*t*↓_, *a*_*t*↓_) states. Second, the two or one hole at the triple-degenerate state of *V*_Zn_ or the *V*_Zn_H complex, respectively, induces the Jahn–Teller effect, which drives a strong nuclear reorganization and symmetry breaking. As a result, the quasi triplet splits into an occupied *a*_*t*↓_ singlet and an empty *e*_*t*↓_ doublet in the case of *V*_Zn_, and an occupied *e*_*t*↓_ doublet and an empty *a*_*t*↓_ singlet in the complex. For the Zn vacancy, these *e*_*t*↓_ and *a*_*t*↓_ states are reflected in the DOS results as depicted in Figure 5a. In particular, in bulk ZnO, the *V*_Zn_–*a*_*t*↓_ and *e*_*t*↓_ states are located about 2.25 and 2.5 eV above the VBM, respectively. The deep character of the zinc vacancy acceptor state is indicated by the calculated 0/–1 transition level, which was determined to be approximately 1.15 eV above the VBM (see Figure 2a), in agreement with the other first-principle calculations^2,7,62^ and experiments.^63^

**Figure 6:**
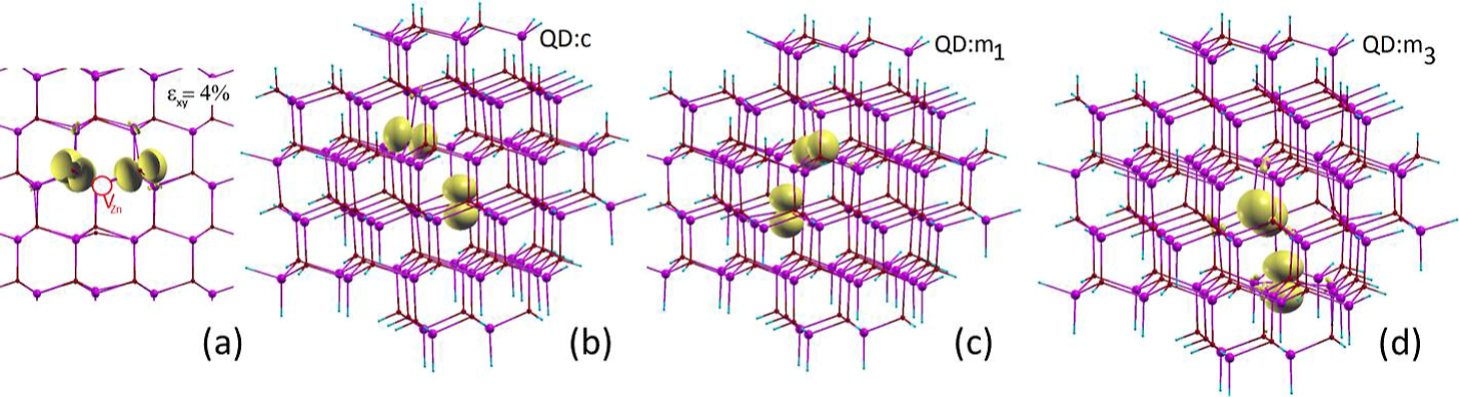
Calculated atomic configurations and isosurfaces of spin density corresponding to 0.05 electron/Bohr^3^ for the neutral zinc vacancy: (a) in the bulk ZnO under 4% compressive strain, (b–d) in QD at *c*, *m*_1_, and *m*_3_ positions, respectively.

The creation of the vacancy or its complex is very sensitive to local atomic perturbations and structure.^1,2,62^ As was previously shown, the energetically lowest solution is due to the previous slight symmetry breaking and possible hole configurations.^1,2,62^ In the ground state, the density of spin polarization of the empty states of zinc vacancy and the complex demonstrates that each hole localizes onto a single sp^4^ orbital of the nearest neighbor O ions, as shown in Figure 6a for *V*_Zn_. The computation results for the nonground state show a very small Δ_*e–a*_ splitting, i.e., the difference between *e*_*t*↓_ and *a*_*t*↓_ energies. This is a direct result of the delocalization error that occurs at lower energies due to dividing the holes between all the nearest neighboring O ions orbitals,^2,64^ which is exemplified in Figure S8. Similar behavior of the vacancy wave functions was obtained within the LDA/GGA approximation.^38,62,64^ Taking the above considerations into account, we break the *V*_Zn_ symmetry by the application of biaxial strain in the bulk crystal and surface proximity in the QD. The strain leads to additional lattice perturbations around the vacancy, which are reflected in its electronic structure and the shape of the spin polarization distribution, as depicted in Figures 5 and 6. In particular, in parallel to the increase of compressive strain from 0 to 4%, the splitting, i.e., the difference between *e*_*t*↓_ and *a*_*t*↓_ energies that is referred in Figure 5a,b as Δ_*e–a*_, increases from 0.25 to 4.7 eV. Moreover, a long tail can be observed in the spin density distribution, partly due to the resonance character of *a*_*t*↓_, which is hybridized with the VBM exhibiting mostly the *p*-nature (see Figure 6a). The impact of surface on the electronic and spin structure of *V*_Zn_ is further confirmed in Figures 5c–e and 6b–d, which show the DOSs and spin density distributions for different positions in the QD. As in the bulk case, here the wave functions of the empty vacancy states are also localized on two sp^4^ dangling bonds, but one of these dangling bonds is directed along the *c*-axis (Figure 6b–d). Generally, this description of the deep vacancy levels in the QD is consistent with the conclusions of bulk calculations because quantum confinement does not highly influence the localization of the levels that remain pinned to their energy, regardless of the nanocrystal size.

**Figure 7:**
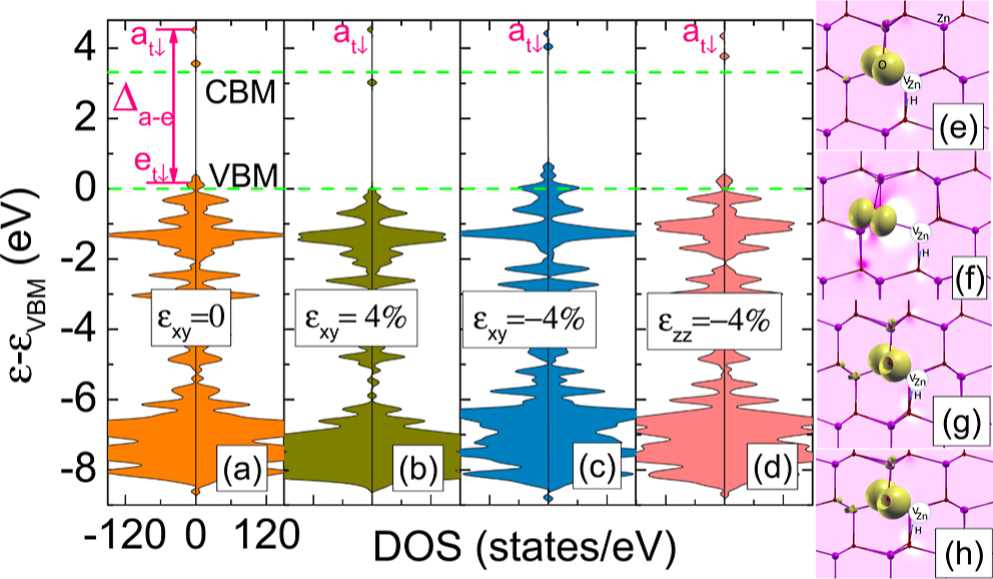
(a–d) Total spin-resolved DOS of ZnO containing a *V*_Zn_H: (a) unstrained, (b) ε_*xy*_ = 4%, (c) ε_*xy*_ = −4%, and (d) ε_*zz*_ = −4%. Left and right panels denote the spin-down and -up channels, respectively. Green dashed horizontal lines correspond to the VBM and CBM unstrained crystal. *a*_*t*↓_, *e*_*t*↓_ levels, and Δ_*a–e*_ are depicted for the unstrained crystal. (e–h) Yellow isosurface represents the spin-density distribution of the ZnO containing a *V*_Zn_H: (e) unstrained, (f) ε_*xy*_ = 4%, (g) ε_*xy*_ = −4%, and (h) ε_*zz*_ = −4%.

The impact of strain on the electronic and spin structure of a complex is depicted in Figure 7a–d, which shows the DOSs for ZnO containing the *V*_Zn_H complex under 0, ε_*xy*_ = 4%, ε_*xy*_ = −4%, and ε_*zz*_ = −4% strain, respectively. Figure 7a shows the total DOS of the complex without strain. It can be seen that the Jahn–Teller effect induces splitting of the quasi triplet into an occupied *e*_*t*↓_ doublet and an empty *a*_*t*↓_ singlet. The splitting Δ_*a–e*_, i.e., in this case the difference between the *a*_*t*↓_ and *e*_*t*↓_ energies is very large and depends on the strain value (see Figure 7). Δ_*a–e*_ is 4.5, 5.0, 3.85, and 4.15 eV for ε_*xy*_ = 0%, ε_*xy*_ = 4%, ε_*xy*_ = −4%, and ε_*zz*_ = −4% strain, respectively. It is worth noting that for the unstrained case, a small atomic displacement was introduced before calculations. The obtained results are accompanied by the spin density calculations for the above configurations (Figure 7e–h). In particular, the results indicate that complex states are dominated by the localized and spin-polarized contributions of the sp^4^ orbitals of the nearest neighbor. The strong localization of the spin density on one O orbital is due to the fact that *a*_*t*↓_ is a deep state. Under compressive strain, the spin density of the complex is more delocalized, which can be observed as a long-range tail that involves *p*(O) orbitals of distant O ions, due to the strong *p*(O)–*d*(Zn) hybridization. It is to be noted that both *p*(O) and *d*(Zn) orbitals build the VBM, and under strain, *p*(O)–*d*(Zn) hybridization will increase.

Atomic displacements around the defect complex under strain affect its electronic structure. After relaxation of the crystal structure in a supercell, the average O–O bond length is 3.195, 3.25, 3.16, and 3.18 Å for ε_*xy*_ = 0%, ε_*xy*_ = 4%, ε_*xy*_ = −4% and ε_*zz*_ = −4% strain, respectively. The change of the strain is accompanied by a strong change of the average O–O bond length around vacancy: 3.289, 3.55, 3.01, and 3.06 Å for ε_*xy*_ = 0%, ε_*xy*_ = 4%, ε_*xy*_ = −4%, and ε_*zz*_ = −4% strain, respectively. These changes include the Jahn–Teller perturbation that is also affected by the applied strain.

### 3.3 Experimental Results

**Figure 8:**
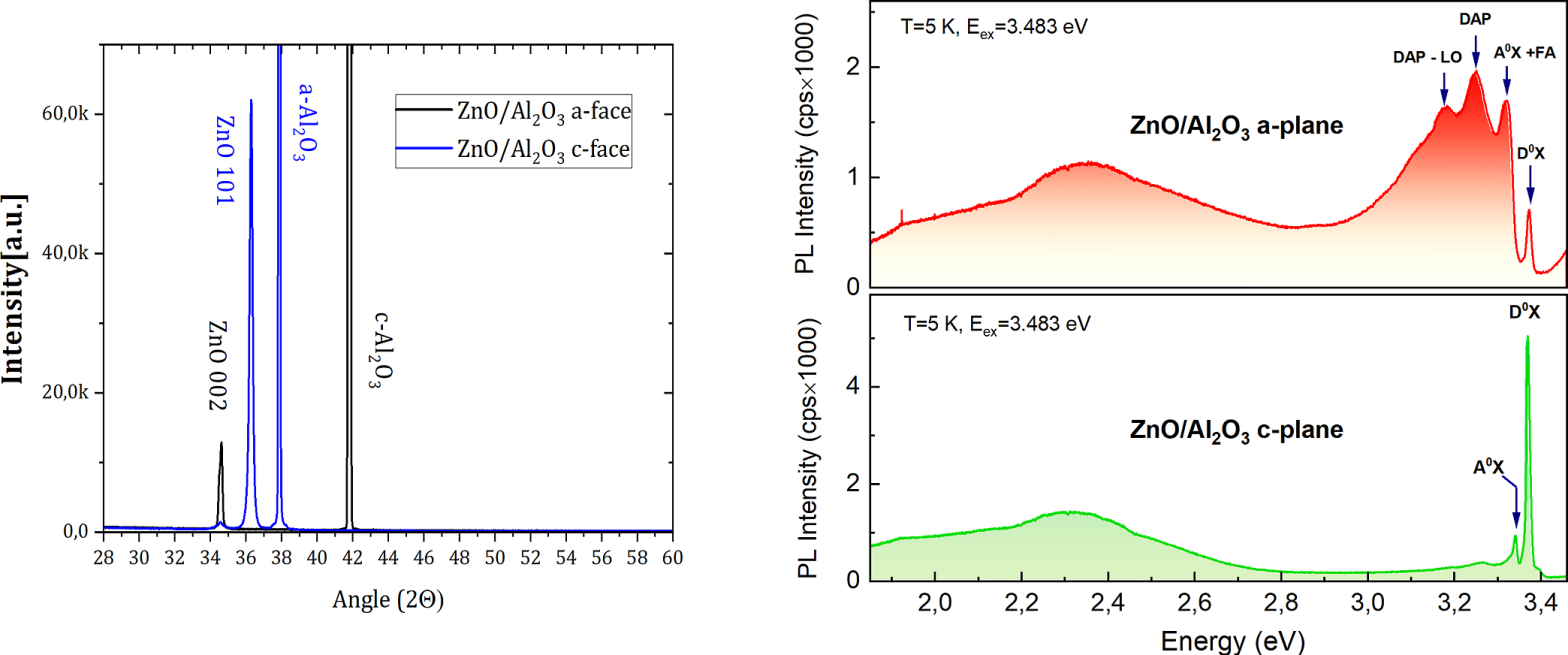
(a) X-ray diffractograms of ZnO films deposited on *a*-Al_2_O_3_ (blue) and *c*-Al_2_O_3_ (black) substrate; (b) photoluminescence spectra of ZnO/*a*-Al_2_O_3_ (up) and ZnO/*c*-Al_2_O_3_ (down) measured at 5K using excitation energy of 3.483 eV.

The X-ray diffractograms revealed a large difference in the crystallographic orientation of both ZnO films, which were grown in the same ALD process on *c*- and *a*-sapphire. The wurtzite-type crystal structure showed a strongly dominant [002] crystallographic direction for the ZnO/*c*-Al_2_O_3_ layer, while a dominant [110] crystallographic direction was accompanied by a minor [002] for the ZnO/*a*-Al_2_O_3_ layer (Figure 8a).

Near-band edge (NBE) photoluminescence of zinc oxide has been the subject of fundamental research over the last 50 years. It has been studied in detail, and the nature of the emission lines is well established. It generally originates from excitonic emission transitions and donor–acceptor pair (DAP) recombination. Free exciton lines (*X*) are located in the range 3.376–3.387 eV, donor-bound exciton lines D^*o*^X are located in the range 3.355–3.375 eV, and acceptor-bound excitons A^*o*^X emit in the range 3.320–3.358 eV.^65,66^ The shallow DAP emission band is located in the range 2.8–3.25 eV and is formed by the zero-phonon line and a number of intensive LO-phonon replicas. In this study, we observe a spectacular difference in the NBE PL emission spectra of ZnO films grown on both substrates. In the case of the ZnO/*c*-Al_2_O_3_ sample, the PL spectrum is dominated by excitonic emission of the D^*o*^X line with an energy of 3.369 eV (Figure 8b, bottom panel), which is accompanied by a weaker A^*o*^X line with an energy of 3.340 eV. In turn, the PL spectrum of the ZnO/*a*-Al_2_O_3_ sample is dominated by acceptor-related emission with a DAP band at 3.26 eV with a well resolved LO- phonon replica at 3.18 eV accompanied by two narrow A^*o*^X and FA lines situated at 3.329 and 3.316 eV, respectively (see Figure S12 in Supporting Information), while the D^*o*^X line at the energy of 3.373 eV is much weaker (Figure 8b, upper panel). The PL peak energies and their interpretation are consistent with data previously reported for ZnO samples grown by ALD under similar conditions.^13,67^

As the ZnO/*c*-Al_2_O_3_ and ZnO/*a*-Al_2_O_3_ films were deposited together in the same ALD growth process, the differences between them can be caused only by their crystallographic structure. Thus, it might be expected that much more intensive acceptor luminescence observed for the ZnO/*a*-Al_2_O_3_ film is related to the film orientation, strain in the layer, and/or the film microstructure. Analysis of the structural properties of both layers reveals the lattice parameter *a* = 3.258 A for the ZnO/*a*-Al_2_O_3_ film and *c* = 5.206 A for ZnO/*c*-Al_2_O_3_ films, which means that the first layer is subjected to tensile strain while the second one is almost relaxed. As was shown by DFT calculations, tensile strain reduces the formation energy of acceptor complexes, although this effect is stronger for compressive strain. On the other hand, in this case, we are dealing with biaxial strain, the effect of which is always more pronounced. Additional information is provided by calculation of the grain size, which in the case of ZnO/*c*-Al_2_O_3_ is approximately 70 nm, i.e., much larger than for the ZnO/*a*-Al_2_O_3_ film (40 nm). Thus, it can be concluded that both strain and surface proximity contribute to the intense acceptor luminescence of the ZnO/*a*-Al_2_O_3_ film; however, theoretical calculations indicate that the latter effect is stronger. It is worth noting that such a significant influence of the crystallographic structure of the thin ZnO films on their shallow donor- and acceptor-related emission is shown for the first time.

## 4 Conclusions

In conclusion, we show the DFT results of a systematic analysis of the impact of different strain conditions and surface proximity on the electronic structure of zinc oxide and the formation energy of acceptor complexes that involve zinc vacancies (*V*_Zn_) and/or –Hx groups. A wide range of strain conditions were considered: tensile and compressive strain, biaxial strain in a planar plane, and uniaxial strain along the *c*-axis. We also considered the QDs crystal to study the importance of surface proximity.

It has been shown that strain noticeably affects the formation energy of acceptor complexes in zinc oxide. This effect might be responsible for the grouping of acceptors, which can be formed only in the crystallites showing compressive strain or near the surface. It was also shown that the presence of hydrogen is responsible for the increased stability, i.e., the reduction of formation energy of the (*V*_Zn_) point defect, which can be further decreased by compressive strain. In support of the DFT results, the PL spectra reveal considerably different intensive acceptor luminescence for the ZnO samples with different crystallographic structures.

The obtained DFT results shed new light on abundant experimental reports showing that *p*-ZnO is much easier to obtain in nanostructures or thin films than in bulk ZnO. There are several reasons for this, such as easier formation of acceptor states and lowering their energy, as well as increased hydrogen incorporation in the near-surface layer.

## Supporting Information Available

Results for DOS of bulk and QD ZnO, DOS of ZnO/H, results of calculations with fixed *a* = *b* for each strain while relaxing the *c*-axis length, geometry of QD, calculated atomic configurations for the neutral vacancy and complex without previously breaking symmetry, and deconvolution of LT PL spectrum from the ZnO/*a*-Al_2_O_3_ film (PDF)
