# Supplementary material for: Effect of Strain and Surface Proximity on the Acceptor Grouping in ZnO
Source: ACS Omega. 2023 Nov 1;8(45):43099–108. doi: 10.1021/acsomega.3c06556 (PMC10652265; doi:10.1021/acsomega.3c06556)
Supplement: Supplementary file 1 — ao3c06556_si_001.pdf [file ao3c06556_si_001.pdf]

Supplementary material

Effect of Strain and Surface Proximity on the Acceptor Grouping in ZnO

Oksana Volnianska*, Vitalii Ivanov, Lukasz Wachnicki, Elzbieta Guziewicz*

Inst. of Physics, Polish Academy of Sciences, Al. Lotników 32/46, 02-668 Warsaw, Poland

*corresponding authors, e-mail: volnian@ifpan,edu.pl, guzel@ifpan.edu.pl

S1. The ZnO bulk without strain and under biaxial strain.

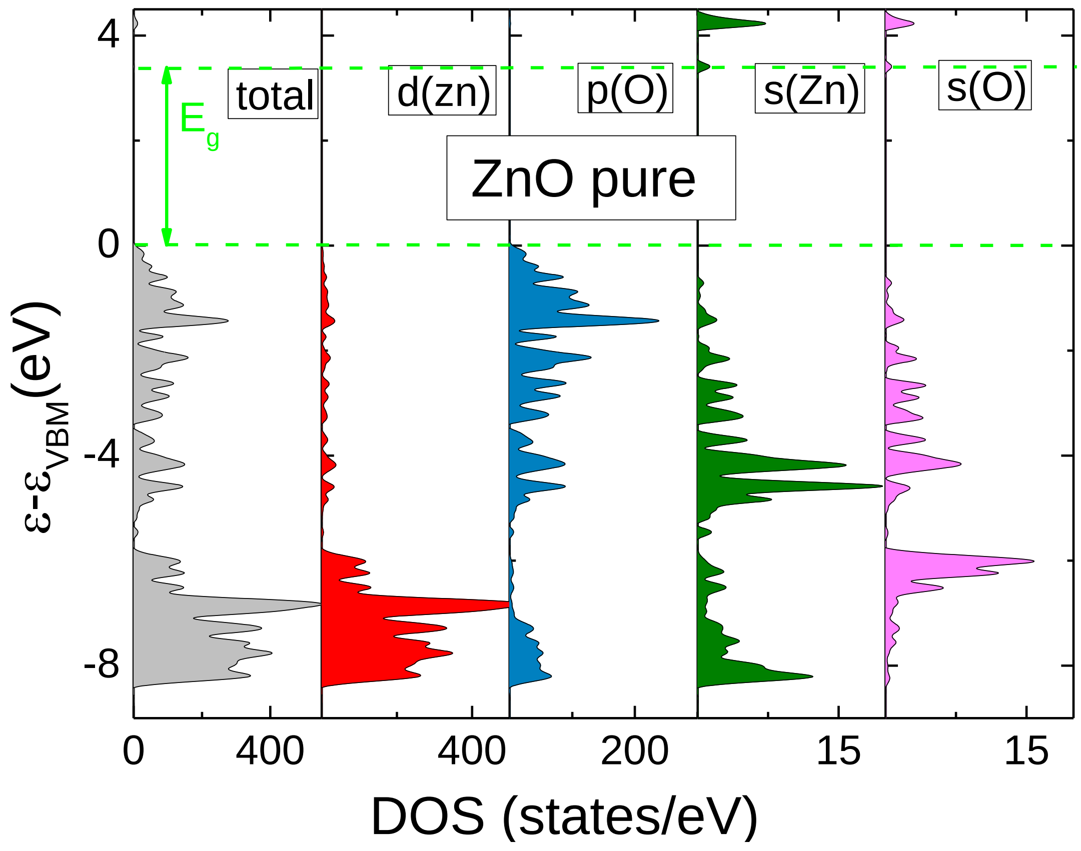

Fig. S1 The calculated total (grey) DOS of bulk ZnO and the summarized contribution of d(Zn) (red color), p(O) (blue), s(Zn) (olive), and s(O) (pink) orbitals, respectively. The 128 -atom super cell results. The valence band maximum (VBM) is built from p(O) with a small contribution of d(Zn) orbitals. The conduction band minimum (CBM) is built mostly from the s(Zn) orbital.

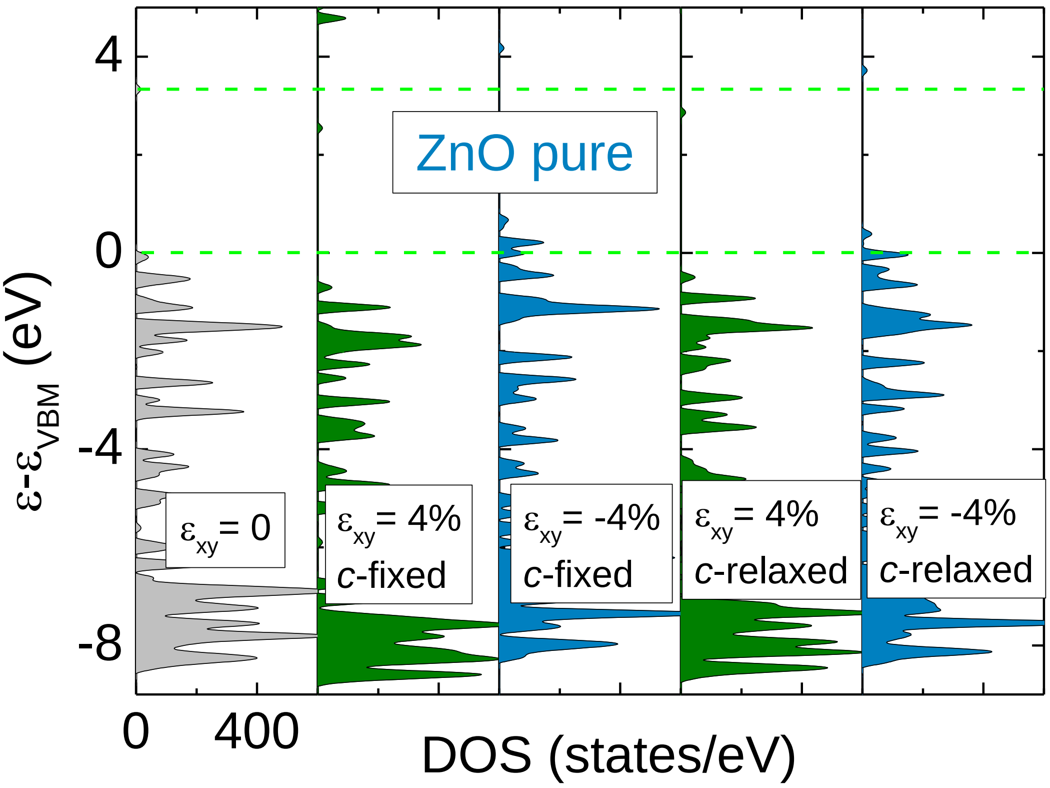

Fig. S2. The calculated DOS of ZnO under 0, 4 % tensile, and -4% compressive biaxial strain, respectively. Calculations were performed with fixed a = b for each strain, while fixing as $c=c_0$ or relaxing the *c*-axis length. The 128 -atom super cell results. Gamma-point.

If a_0, c_0 are the lattice constants of the perfect wurtzite ZnO crystals, then the biaxial strain is defined as $\varepsilon_{xy} = (a-a_0)/a_0 \times 100$ %, where a is the lattice constant of ZnO in its strained state. During the calculations, the lattice parameters were kept fixed at the specified strained a = b and $c=c_0$ and the internal coordinates of atoms were relaxed. Additionally, considering the substrate clamping effect, we performed some calculations with fixed a = b for each strain, while relaxing the c-axis length, however, in both cases the results were qualitatively similar.

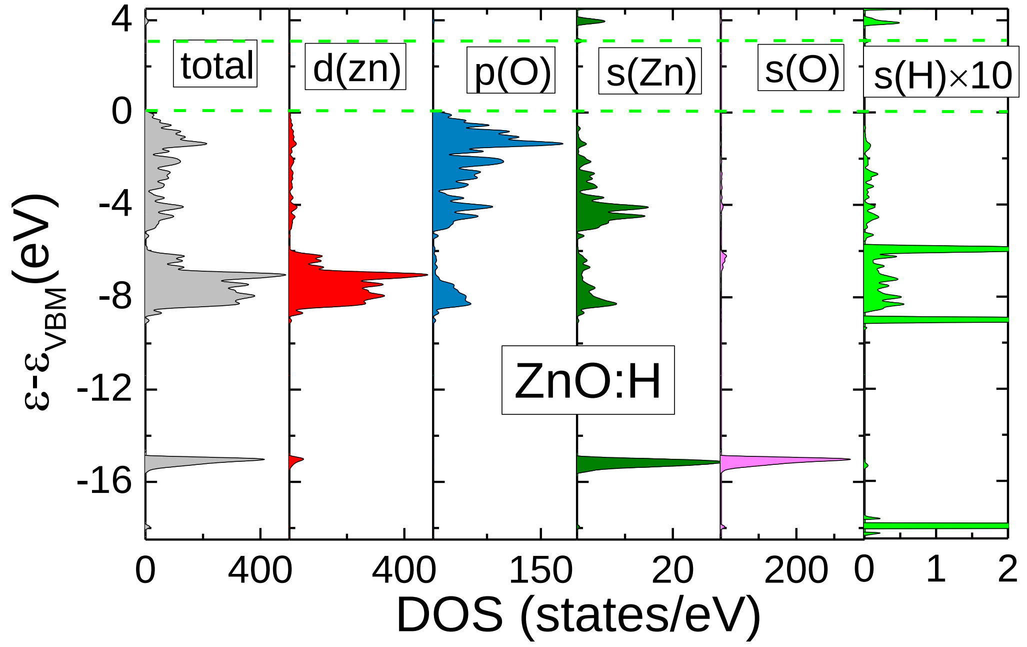

Fig. S3 The calculated total (grey) DOS of unstrained $ZnOH_i$ and the summarized contribution of d(Zn) (red color), p(O) (blue), s(Zn) (olive), s(O) (pink), and s(H)*10 orbitals, respectively.

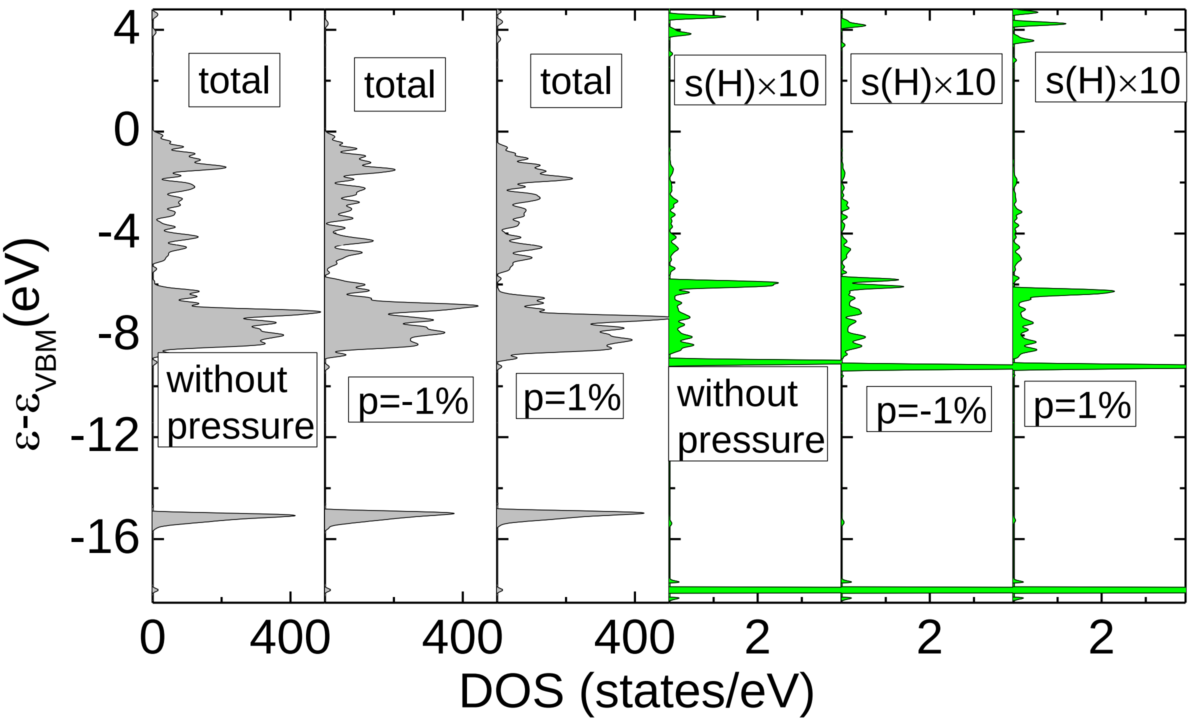

Fig. S4 The calculated total (grey) DOS of $ZnOH_i$ and s(H)*10 orbitals without, -1% compressive, and 1% tensile hydrostatic pressure, respectively. The 128 -atom super cell results.

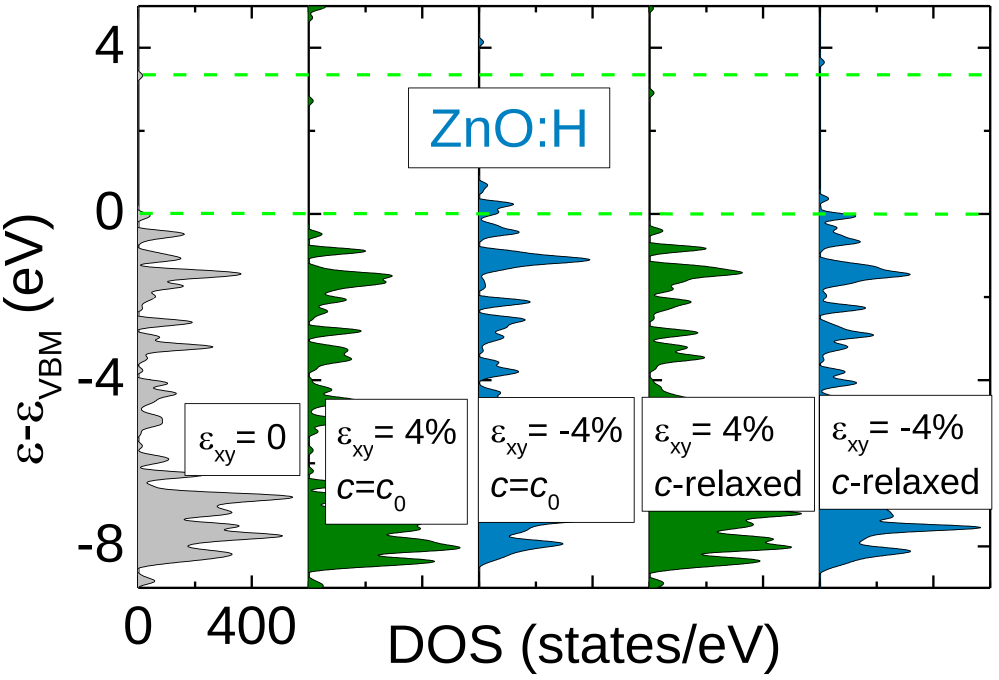

Fig. S5. The calculated DOS of ZnO:H under 0, 4 % tensile, and -4% compressive biaxial strain, respectively. Calculations were performed with fixed a = b for each strain, while fixing as $c=c_0$ or relaxing the *c*-axis length. The 128 -atom super cell results. Gamma-point.

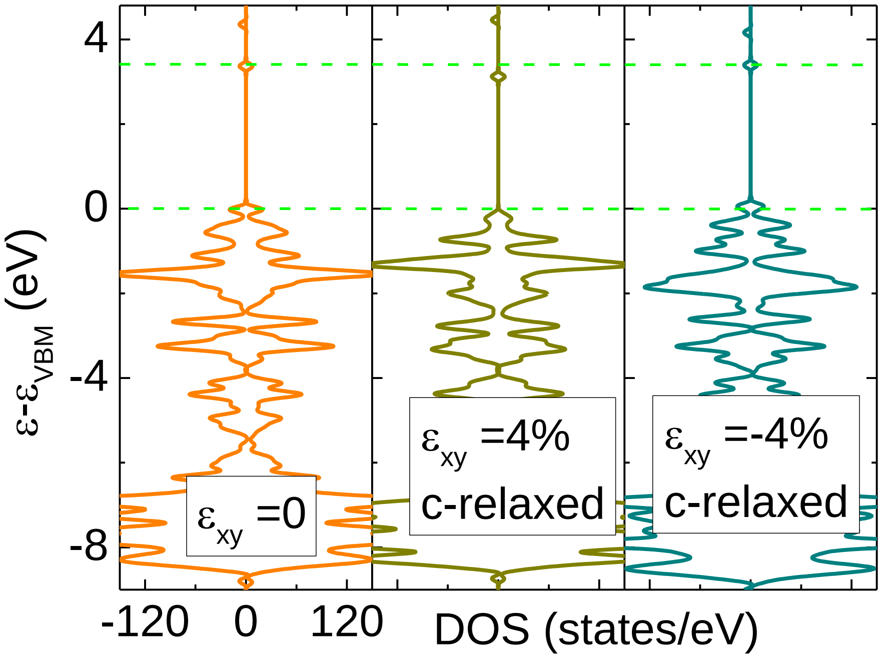

Fig. S6. The calculated DOS of ZnO:V_{-Zn}H under 0, 4 % tensile, and -4% compressive biaxial strain, respectively. Calculations were performed with fixed a = b and *c*-axis length was relaxed.

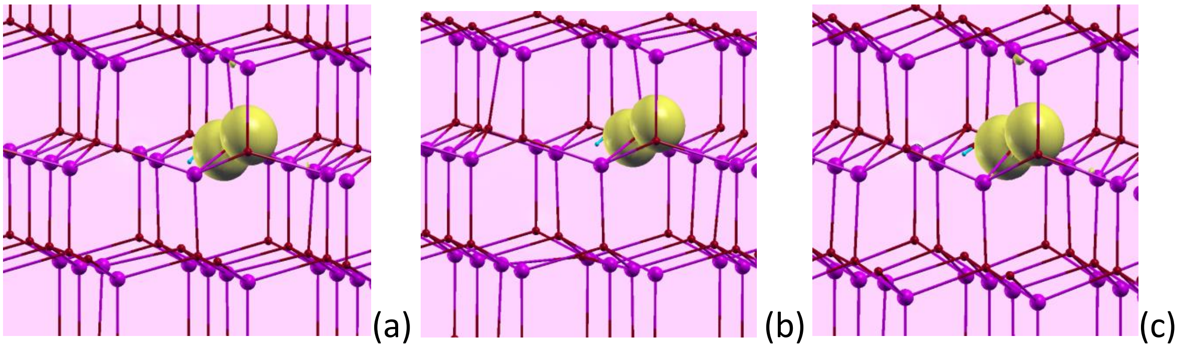

Fig. S7. Calculated atomic configurations and isosurfaces of spin density corresponding to 0.05 electron/bohr^3 for the neutral $V_{Zn}H$ complex: (a) without strain, (b), (c) under -4% and 4% strain.

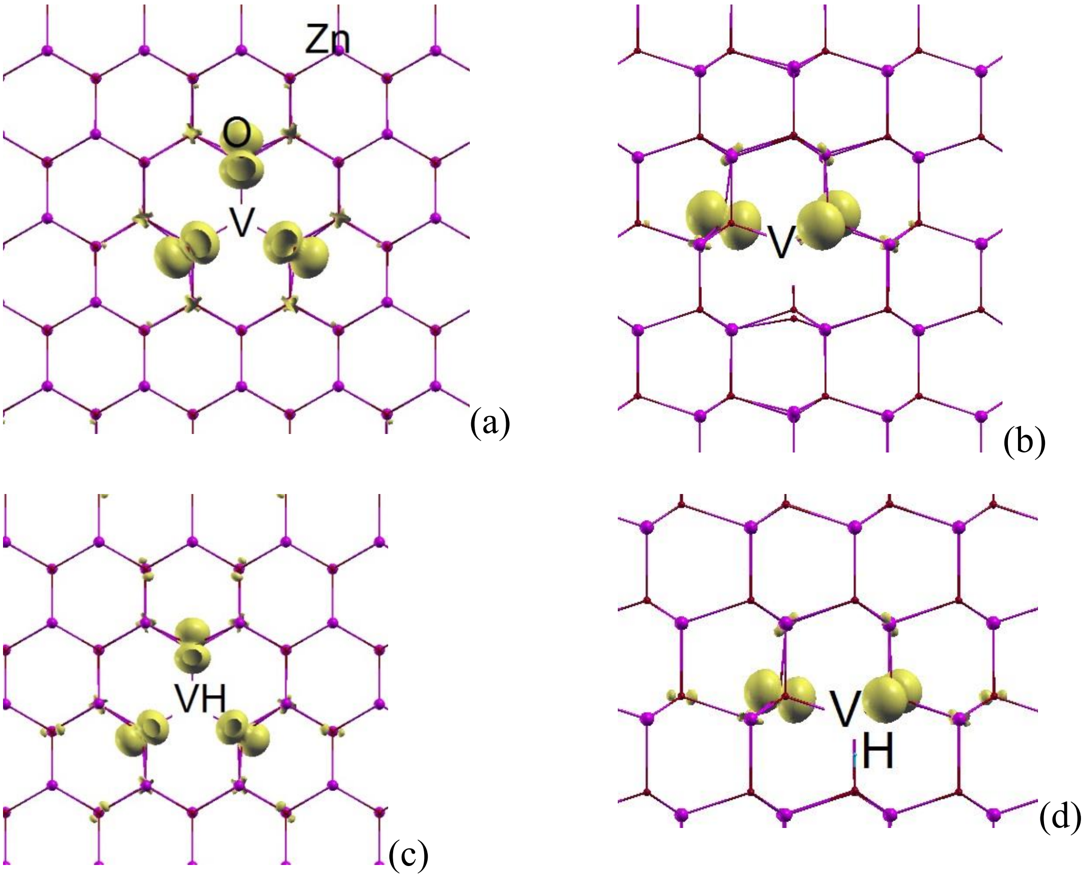

Fig. S8. Calculated atomic configurations and isosurfaces of spin density corresponding to 0.05 electron/bohr^3 for the neutral V_{Zn} (a), (b) and the $V_{Zn}H$ complex (c), (d) without previously breaking symmetry. (a), (c) image in the xy-plane, (b), (d) image along the c-axis. The spin density of two (one) holes is delocalized on the three (two) dangling bonds and spin density distributions include long tails involving far neighbors of the vacancy. Similar delocalized vacancy wave functions was observed under the LDA/GGA approach, see for example [1].

S2. The ZnO QDs.

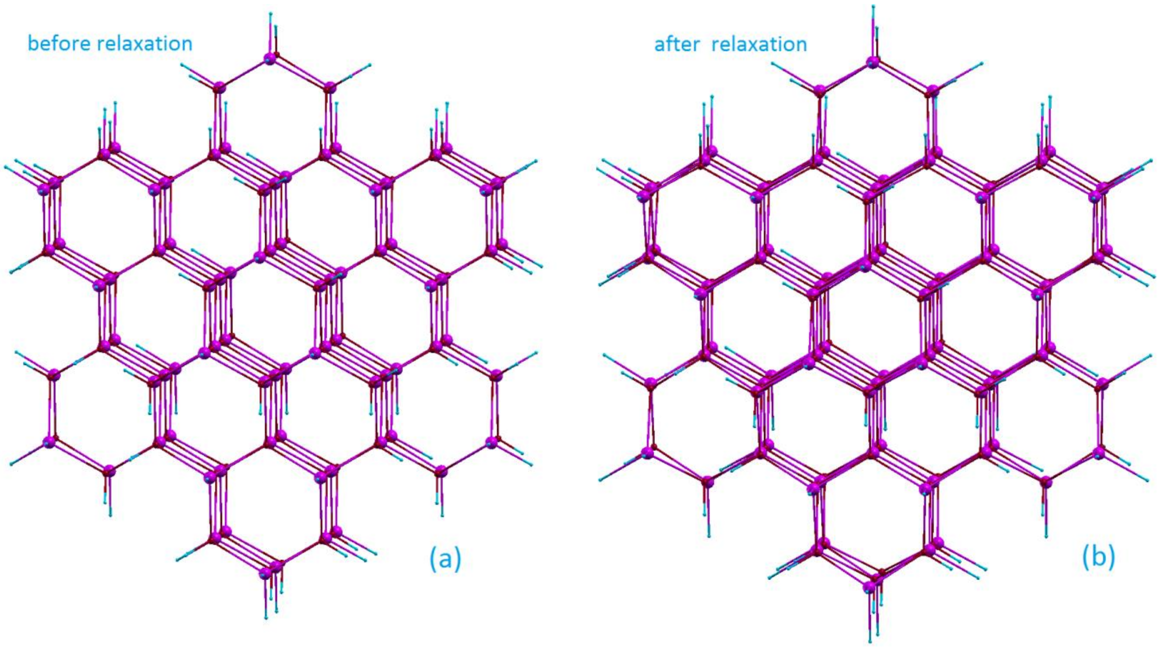

Fig. S9. The structure ZnO QD: (a) before and (b) after ionic relaxation. The Zn atoms are drawn with large spheres (purple color), the O atoms with medium spheres (red), and the pseudohydrogens with small spheres (blue).

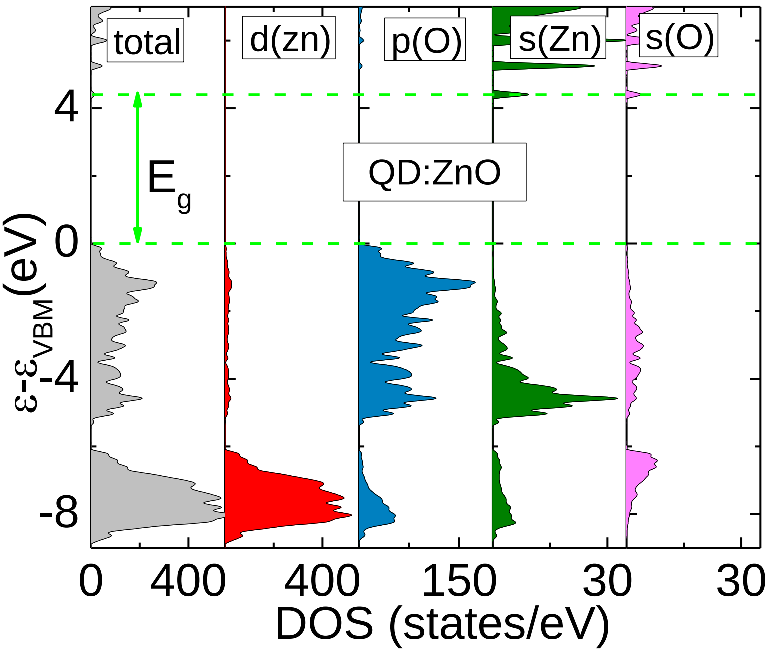

Fig. S10. The calculated total (grey) DOS of ZnO QD and the summarized contribution of d(Zn) (red color), p(O) (blue), s(Zn) (olive), and s(O) (pink) orbitals, respectively. The valence band maximum (VBM) is built from p(O) with a small contribution of d(Zn) orbitals. The conduction band minimum (CBM) is built in mostly from s(Zn) and s(O) orbitals.

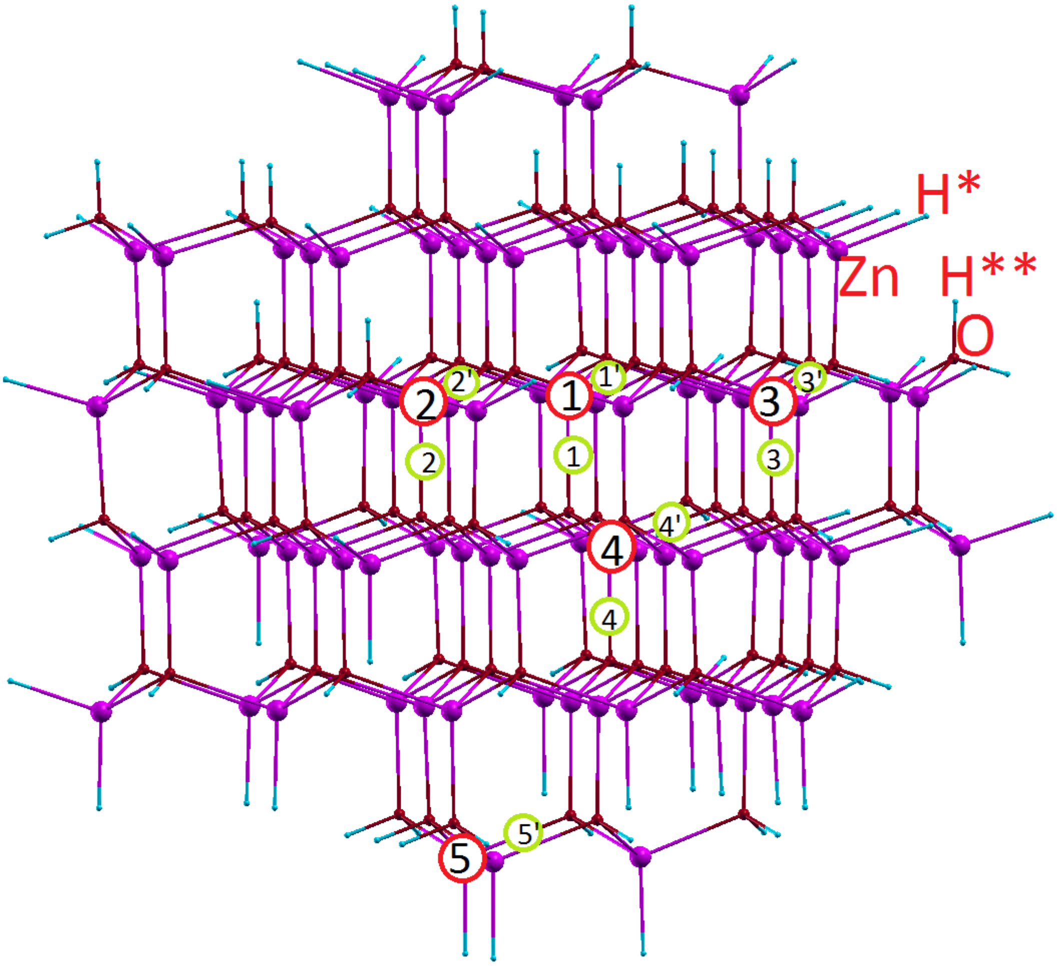

Fig. S11. The structure of ZnO QD after ionic relaxation. The numbers 1 -- 5 in the red circle denote Zn at 1: c, 2: m_1, 3: m_2, 4: m_3, and 5: m_4 sites, respectively. The numbers 1' – 5' denote the hydrogen positions at the center of bonds in the *xy*- plane. The numbers 1 – 4 denote the hydrogen positions at the center of bond along c-axis. The H atom at sites labeled as 1 – 4 and 5' correspond to the c, m_1, m_2, m_3 and m_4 configurations in the main text.

S3. Photoluminecence spectra of ZnO.

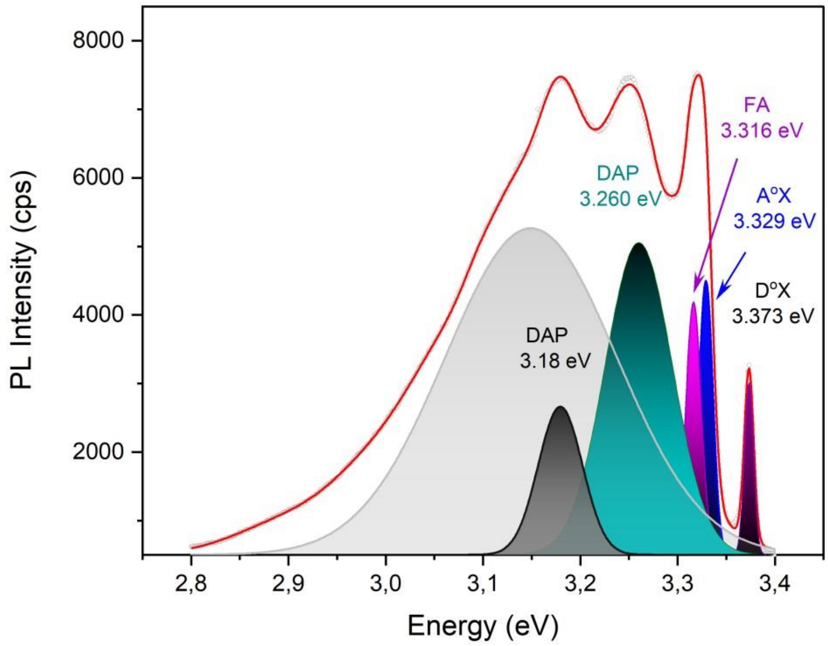

Fig. S12 Deconvolution of the band-edge PL of the ZnO/a-Al2O3 film

----

1. *O. Volnianska, P. Boguslawski, High spin states of cation vacancies in GaP, GaN, AlN, BN, ZnO and BeO: A first principles study. Phys. Rev. B 83, 205205 (2011).*
